# Design and Force/Angle Independent Control of a Bionic Mechanical Ankle Based on an Artificial Muscle Matrix

**DOI:** 10.3390/biomimetics9010038

**Published:** 2024-01-06

**Authors:** Zhikun Jia, Guangming Han, Hu Jin, Min Xu, Erbao Dong

**Affiliations:** CAS Key Laboratory of Mechanical Behavior and Design of Materials, Department of Precision Machinery and Precision Instrumentation, University of Science and Technology of China, Hefei 230026, China; jzk1997@mail.ustc.edu.cn (Z.J.); hgm@mail.ustc.edu.cn (G.H.); jhrdsp@ustc.edu.cn (H.J.); xumin@ustc.edu.cn (M.X.)

**Keywords:** shape memory alloy, artificial muscle, bionic ankle, matrix control

## Abstract

Inspired by the natural skeletal muscles, this paper presents a novel shape memory alloy-based artificial muscle matrix (AMM) with advantages of a large output force and displacement, flexibility, and compactness. According to the composition of the AMM, we propose a matrix control strategy to achieve independent control of the output force and displacement of the AMM. Based on the kinematics simulation and experiments, we obtained the output displacement and bearing capacity of the smart digital structure (SDS) and confirmed the effectiveness of the matrix control strategy to achieve force and displacement output independently and controllably. A bionic mechanical ankle actuated by AMM was proposed to demonstrate the actuating capability of the AMM. Experimental results show that the angle and force of the bionic mechanical ankle are output independently and have a significant gradient. In addition, by using a self-sensing method (resistance self-feedback) and PD control strategy, the output angle and force of the bionic mechanical ankle can be maintained for a long time without overheating of the AMM.

## 1. Introduction

Skeletal muscles are the driving power of the human motion system with abilities of actuating, self-sensing, exploding and energy-storing [1]. In recent decades, a large number of scholars have conducted, and are still conducting, research on imitating the form and function of skeletal muscles. Different types of actuators have been put forth and extensively studied for their potential applications as “artificial muscles” [2], including traditional actuator devices (such as electric motors [3,4], hydraulic actuators [5,6,7,8] and pneumatic actuators [9,10,11,12]) and smart materials (such as electroactive polymers [13,14,15,16], high-strength polymer fibers [17], magnetostrictive alloys [16] and shape memory alloys [1,2,18,19,20]).

Electric motors are limited to imitate skeletal muscles as they usually need to work with complex transmission mechanisms and gear systems that can significantly increase mass and reduce energy density. Similarly, hydraulic actuators also suffer from low energy density and additional hydraulic pumps and valves will complicate the entire system and simultaneously bring a variety of sealing problems. Pneumatic artificial muscles, such as the “McKibben Muscles”, in particular are intrinsically compliant and can thus mimic some of the properties of natural muscles. However, these systems require air compressors that are neither light nor small. In addition, the actuators mentioned above will generate noise during working and cannot imitate the self-sensing capability of skeletal muscles without additional sensors [16]. Recently, electroactive polymers (EAPs) are attractive for a wide range of applications as artificial muscles for reasons such as their large active strains, no acoustic noise, and self-sensing ability. Unfortunately, the generated force of an EAP artificial muscle is small and the input voltage is too high (>1000 V) to be of practical use [15,21,22]. High-strength polymer fibers have the advantages of fast speed, large displacement, and the ability to withstand a greater load, which has a wide range of potential applications. However, low energy density is a common issue for high-strength polymer fibers. In addition, due to the fact that high-strength polymer fibers usually require complex deformation such as bending, stretching, or twisting, fatigue failure may occur during long-time use. This means that the fibers may break down over time [17]. Magnetostrictive alloys (MAs) have some excellent properties such as large magnetostrictive strain, high energy density and strong propelling force. However, the magnetostrictive effect exhibits notable nonlinear characteristic and frequency-dependent hysteresis. Moreover, the devices made with MAs usually work in an alternating magnetic field, which can cause mechanical energy loss, thus reducing the energy conversion efficiency of MAs during operation [23]. 

Comparing with the actuators mentioned above, shape memory alloys (SMAs) act like a natural muscle because of their high energy density, strong load capacity, quick response, silent operation, flexibility and self-sensing abilities [1,2,18,24]. Moreover, SMAs can be actuated using resistive heating by low voltage, which is easier to achieve. These capabilities make the SMAs promptly devote to soft wearable robots [20], morphing aircraft structures [25] and bionic soft robots [26,27,28].

Natural skeletal muscles are composed of numerous contraction-capable muscle cells (also called muscle fibers) which are covered and maintained by connective tissues. The muscle fibers contain a number of continuously arranged sarcomeres that are the basic structural units of skeletal muscle contraction [29]. The number of sarcomeres determines the ability of muscle contraction and the number of muscle fibers determines the strength of the muscle. Inspired by the composition of natural skeletal muscles, this paper proposed an artificial muscle matrix (AMM) using SMA wires as the muscle fibers. The AMM contains a number of smart digital structures (SDSs) in series which are exactly imitation of the sarcomeres in natural muscles. The SDSs are wrapped by silicone (Ecoflex 00-30) that plays the role of protection and maintenance just like the connective tissues in natural muscles. And in each SDS, there are many SMA wires arranged in parallel, which is similar to the constitution mechanism of natural skeletal muscles. The number of SDS, *m*, determines the contraction displacement of the AMM and the number of SMA wires in each SDS, *n*, determines the output force of the AMM. Any combination of *m* and *n* will result in different force/displacement output of the AMM. Obviously, the greater of *m* and *n*, the more situations (m × n) of the force/displacement output. 

The goal of this research is to develop a novel SMA-based AMM which can be applied into various bionic joints. The AMM not only has the abilities of the large force and displacement, flexibility, light, silence, and compactness, but also can achieve the synchronization control of the output force and displacement easily. Furthermore, the self-sensing capability is achieved by exploiting the variation in the electrical resistance of the SMAs during actuation to control the temperature of the SMA wires and hence prevent their overheating, and hence enhanced the performance of the AMM. In this paper, we made a demo version (both *m* and *n* are 4) of the AMM and applied the AMM to a homemade bionic mechanical ankle to demonstrate the AMM actuating capability. Experimental results demonstrate that the angle and force of the bionic mechanical ankle are output independently and have a significant gradient, which means that the independent control of the angle and force can be achieved. In addition, by a using self-sensing method (resistance self-feedback) and PD control strategy, the output angle and force of the bionic mechanical ankle can be maintained for a long time without overheating of the AMM.

## 2. Design and Fabrication of the Smart Digital Structure (SDS)

**Figure 1:**
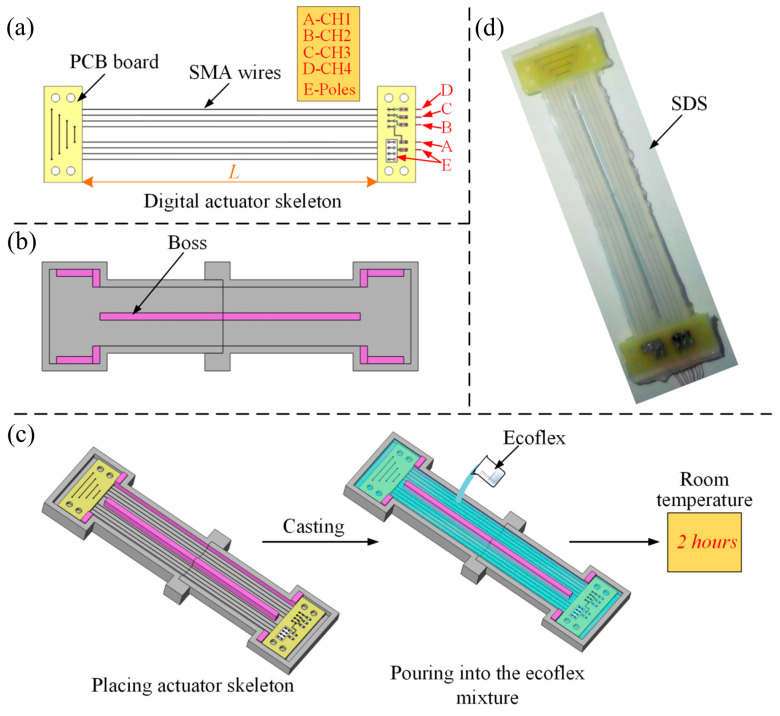
Design and fabrication of the SDS. (**a**) The digital actuator skeleton composed of the SMA wires and PCBs. (**b**) Mold with special boss. (**c**) Procedure of the casting technology. (**d**) The unmolded SDS.

The SDS includes a digital actuator skeleton and a soft body made of ecoflex 00-30 that is a softer-than-skin silicone rubber with high strength and stretch. As shown in Figure 1a, the digital actuator skeleton is composed of two fixed plates and four sets of SMA wires (diameter 0.15 mm and transition temperature 70 °C, produced by DYNALLOY Inc., Irvine, CA, USA) with respect to four channels CH1, CH2, CH3 and CH4, respectively. And all the channels share the poles E. The mold casting process was used to fabricate the SDS whose mold was produced by 3D printer, as shown in Figure 1b. The purpose of the boss in the middle of the mold is to reduce the hindrance of the excess silicone rubber to the linear contraction of the SDS. Figure 1c shows the procedure of the casting technology. Firstly, the digital actuator skeleton was placed in the casting mold. Then, casting the ecoflex mixture (mixing ratio 1A:1B by weight or volume) into the mold to totally encapsulate the digital skeleton. It is worth noting that the liquid silicone mixture before and after pouring needs to be put into a vacuum pump to remove the bubbles. After solidification at room temperature for two hours, the SDS is obtained. 

We used ecoflex 00-30, which plays a role in insulation and protection as the soft underlying body of the SDS and printed circuit boards (PCBs) to be the fix plates. The effective length of the SDS is 80 mm, as L shows in Figure 1a. The totally length and width of the SDS are 95 mm and 25 mm, respectively. The height of the SDS is variable and can be regulated by redesigning the molds. In this paper, all the SDSs applied a height of 2.4 mm.

## 3. Method

### 3.1. Modeling of the SDS

A model is developed to obtained the shrink ability of the SDS. The simulation model is a traditional SMA-based bias spring actuator [30]. And the parameters of the model are listed in Table A1.

As for the bias spring actuator, when the SDS shrinks under contracting force $F_{sma}$ , it will satisfy the external force balance equation:

(1) $$n_{1}\cdot F_{sma}=kL_{0}\left(\varepsilon_{r}-\varepsilon_{sma}\right)$$

where $n_{1}$ is the number of the SMA wires of the SDS, k represents the stiffness of the bias spring, $L_{0}$ is the length of each SMA wire, $\varepsilon_{r}$ is the maximum residual strain of the SMA wire and $\varepsilon_{sma}$ is the strain of the SMA wire.

In addition, each SMA wires follows the equilibrium state equation:

(2) $$F_{sma}=\sigma_{sma}A_{sma}-F_{0}$$

where $\sigma_{sma}$ is the stress in the SMA wire, $A_{sma}$ is the cross-sectional area of the SMA wire and $F_{0}$ is the initial tension of the SMA wire.

According to Equations (1) and (2), the resulting stress of the SMA wire can be expressed by

(3) $$\sigma_{sma}=k_{t}\left(\varepsilon_{r}-\varepsilon_{sma}\right)+\sigma_{0}$$

where $\sigma_{0}=F_{0}/A_{sma}$ and $k_{t}=kL_{0}/n_{1}A_{sma}$ .

When the temperature of the SMA wire heated by voltage is higher than austenite transformation start temperature $T_{s}^{A}$ , the SMA wire starts to transfer from the martensite phase to the austenite phase and generates the contraction force. The basic governing equation from Liang-Rogers’ model is used to describe the behavior of the SMA wire as

(4) $${\dot{\sigma }}_{sma}=E\left(\xi \right){\dot{\varepsilon }}_{sma}+\theta \dot{T}+\Omega \left(\xi \right)\dot{\xi }$$

where E(ξ) is the Young’s modulus of the SMA wire, θ is the thermoelastic tensor of the SMA wire and is assumed to be negligible, T is the temperature of the SMA wire, Ω(ξ) is the phase transformation tensor and ξ is the martensite fraction of the SMA wire. It is generally assumed that E(ξ) is in a linear relationship with the martensite fraction, namely, $E\left(\xi \right)=E_{A}+\xi \left(E_{M}-E_{A}\right)$ , where $E_{M}$ and $E_{A}$ are Young’s modulus when martensite is 100% and austenite is 100%, respectively. Ω(ξ) is related to E(ξ) and can be expressed as $\Omega \left(\xi \right)=-\varepsilon_{r}E\left(\xi \right)$ . Therefore, the basic governing equation can be simplified as

(5) $${\dot{\sigma }}_{sma}=E\left(\xi \right)\left({\dot{\varepsilon }}_{sma}-\varepsilon_{r}\dot{\xi }\right)$$

During the transformation process of the SMA wire from martensite phase to the austenite phase, the martensite fraction can be expressed by

(6) $$\xi =\frac{\xi_{0}}{2}\cos\left[a_{A}\left(T-T_{s}^{A}\right)+b_{A}\sigma_{sma}\right]+\frac{\xi_{0}}{2},\left(T_{s}^{A}+\frac{\sigma_{sma}}{C_{A}}\right)\leq T\leq \left(T_{f}^{A}+\frac{\sigma_{sma}}{C_{A}}\right)$$

where $a_{A}=\pi /T_{f}^{A}-T_{s}^{A}$ and $b_{A}=-a_{A}/C_{A}$ . $\xi_{0}$ is the initial martensite fraction of the SMA wire, $C_{A}$ represents the effect of stress on the austenite temperature of the SMA wire and it is a constant.

When the SMA wire is heated by voltage, its thermal model can be expressed as

(7) $$\rho_{sma}c_{sma}V_{sma}\dot{T}=i_{sma}^{2}R_{sma}-hS_{sma}(T-T_{0})+\rho_{sma}V_{sma}H\dot{\xi }$$

where $\rho_{sma}$ is the density of the SMA wire, $c_{sma}$ is the specific heat capacity, $V_{sma}$ is the volume of the SMA wire, $i_{sma}$ is the supply current, $R_{sma}$ is the resistance of the SMA wire at room temperature, h is the heat transfer coefficient, $S_{sma}$ is the surface area of the SMA wire, $T_{0}$ is the ambient temperature, H is the latent heat of transformation of the SMA wire.

The following equations are derived from Equations (3)–(7).

(8) $$\left[\begin{array}{l} \dot{\xi }\\ \dot{T}\\ \dot{\varepsilon } \end{array}\right]=\left[\begin{array}{l} \frac{\lambda_{T}\frac{i_{sma}^{2}R_{sma}-hS_{sma}\left(T-T_{0}\right)}{\rho_{sma}V_{sma}c_{sma}}}{1+\lambda_{\sigma }k_{t}\varepsilon_{r}\frac{E\left(\xi \right)}{E\left(\xi \right)+k_{t}}-\lambda_{T}\frac{H}{c_{sma}}}\\ \frac{i_{sma}^{2}R_{sma}-hS_{sma}\left(T-T_{0}\right)}{\rho_{sma}V_{sma}c_{sma}}+\frac{H\dot{\xi }}{c_{sma}}\\ \frac{E\left(\xi \right)\xi_{r}}{E\left(\xi \right)+k_{t}}\dot{\xi } \end{array}\right],\left(T_{s}^{A}+\frac{\sigma_{sma}}{C_{A}}\right)\leq T\leq \left(T_{f}^{A}+\frac{\sigma_{sma}}{C_{A}}\right)$$

where $\lambda_{T}=-a_{A}\frac{\xi_{0}}{2}\sin\left[a_{A}\left(T-T_{s}^{A}\right)+b_{A}\sigma_{sma}\right]$ , $\lambda_{\sigma }=-b_{A}\frac{\xi_{0}}{2}\sin\left[a_{A}\left(T-T_{s}^{A}\right)+b_{A}\sigma_{sma}\right]$ .

**Figure 2:**
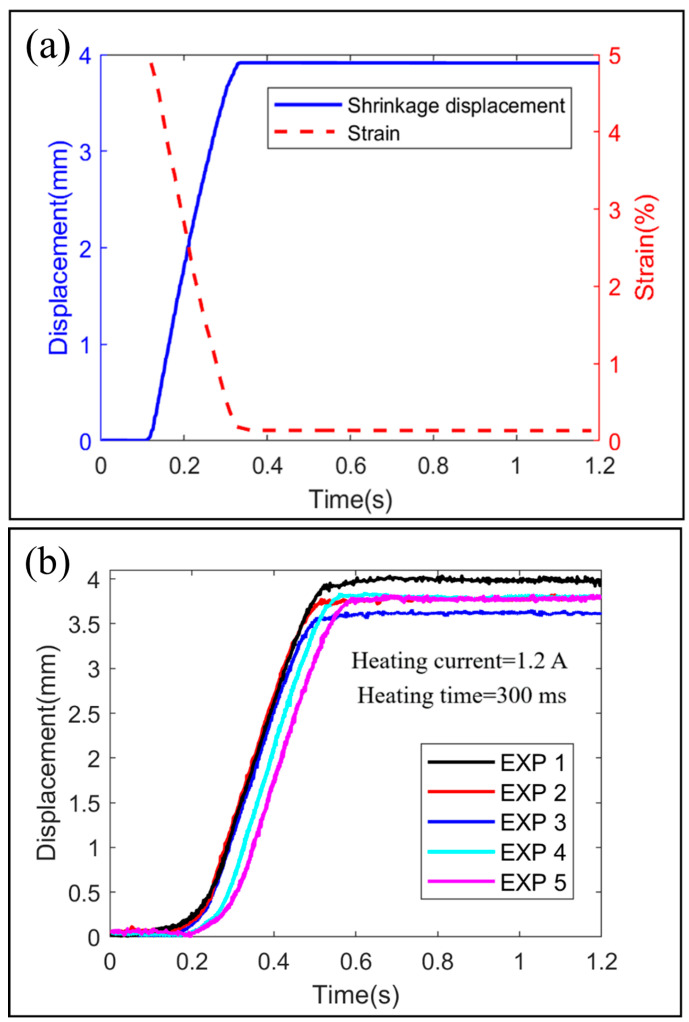
Simulation and experimental results. (**a**) Curves of shrinkage displacement and strain over time in simulation. (**b**) Curves of shrinkage displacement over time of 5 experiments.

The simulation and experimental results that are the curves of shrinkage displacement over time are showed in Figure 2a,b, respectively. It is easy to see that the simulation results are in good agreement with the experimental results, which confirms the effectiveness of the simulation model. The output displacement of the SDS is about 4 mm which means that the deformation of the SDS is about 5% due to the effective length of SDS is 80 mm.

### 3.2. Shrinkage Performance under Constant Loads

**Figure 3:**
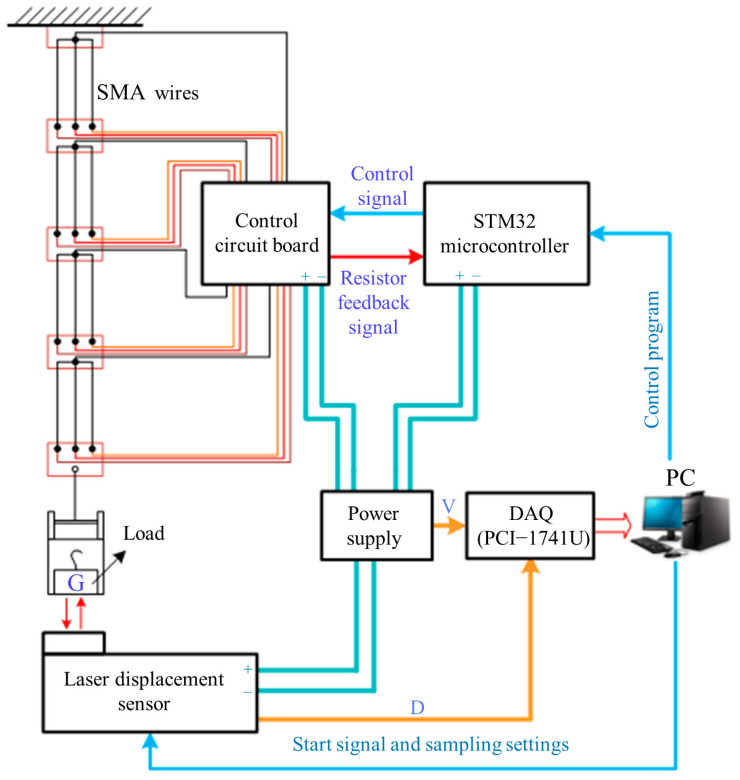
Schematic diagram of experiment setup.

In order to obtain the shrinkage displacement performance of SMA wires under different constant loads, the electro-thermomechanical experiments were carried out, as shown in Figure 3. The SMA wires with diameter of 0.15 mm and length of 80 mm were arranged in parallel (three SMA wires) or in series (four sections) and connected to different constant loads. A leaser displacement sensor with resolution 0.001 mm was used to measure the SMA wires length change. We applied inner heating method by using resistive heating with free convection. The heating current generated from control circuit board which is actually the current amplifier and metal oxide semiconductor switching was used to drive the SMA wires. A computer equipped with a data acquisition card (PIC-1741U) was used to store and process all the measured data.

**Figure 4:**
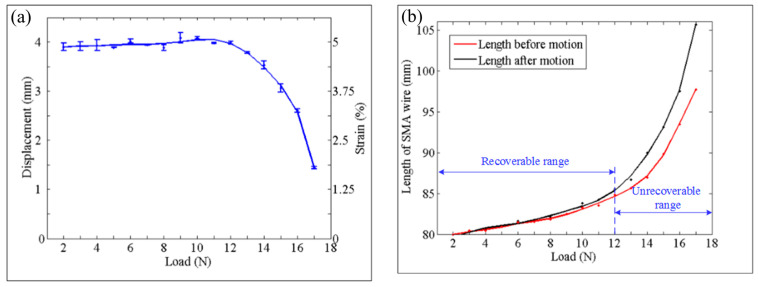
Experimental results. (**a**) Shrinkage displacement and strain of one SMA wire under different constant loads. (**b**) Comparison of the SMA wire length before and after motion.

**Figure 5:**
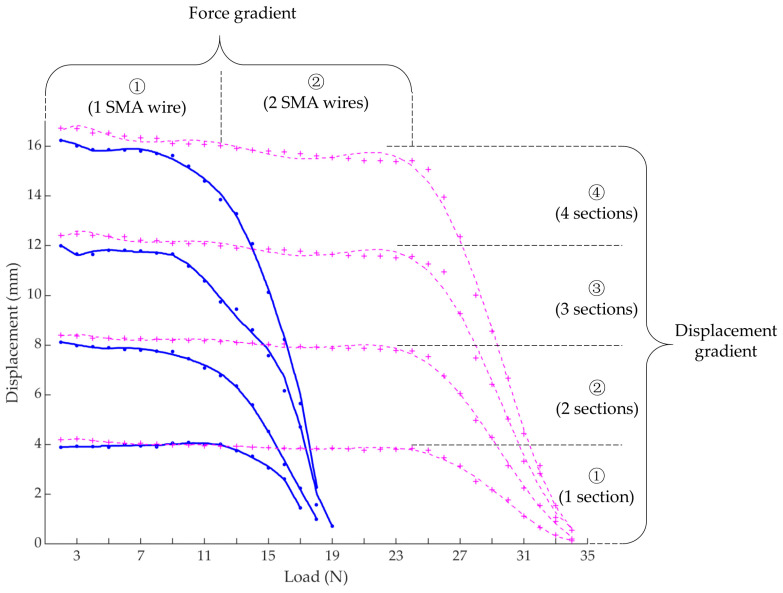
Shrinkage displacement under different constant loads of different number of SMA wires in different number of sections. (in blue) and (in magenta) are cases when only one SMA is actuated and two SMAs are actuated simultaneously, respectively.

Experiments of one SMA wire in one section were firstly carried out to obtain the relationship between shrinkage displacement and different loads. From the results, we can see that the shrinkage displacement of the SMA wire varies little when the load is not greater than 12 N, as shown in Figure 4a. And, under this condition, the length of the SMA wire is compared before and after heating and cooling, it is found that the length of the SMA wire before and after motion also varies little, that is, the SMA wire is recoverable, as shown in Figure 4b. However, when the load exceeds 12 N, the shrinkage capacity decreases significantly and the SMA wire length before and after motion has a larger difference which is unrecoverable under the influence of the excessive loads. In order to further explore the shrinkage performance under different constant loads, experiments of different number of SMA wires in different number of sections were followed. As shown in Figure 5, the trend of each curve of different situations is similar to the curve of one SMA wire in one section which we have described above. In addition, the force gradient can be obtained by driving different number of SMA wires which are arranged in parallel and the displacement gradient can be obtained by driving different number of sections which are connected in series. These also demonstrate the effectiveness of the matrix control method of the artificial muscles to achieve force/displacement output independently and controllably.

## 4. Results

### 4.1. Experimental Setup

**Figure 6:**
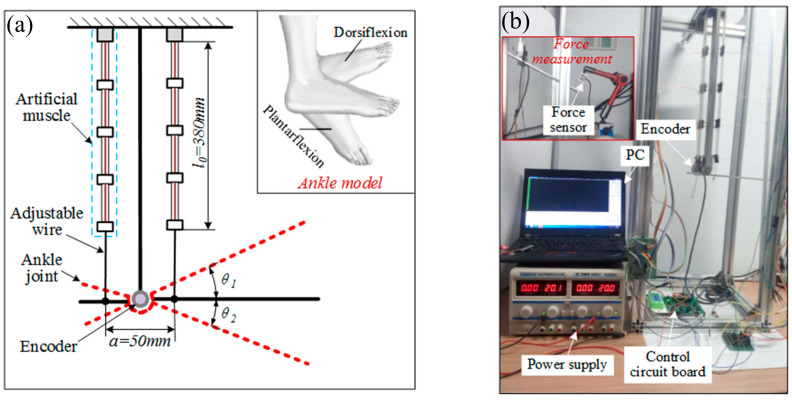
Artificial muscle matrix actuated the bionic mechanical ankle. (**a**) Schematic diagram of bionic ankle. (**b**) Bionic ankle experimental device.

To demonstrate the AMM actuating capability, we applied the AMM to a bionic mechanical ankle, as shown in Figure 6. Like the real ankle, the two artificial muscle matrices are symmetrically distributed on both sides of the joint to achieve dorsiflexion and plantarflexion. A rotary encoder whose model is E6B2-CWZ1X (produced by Omron with a resolution of 1000 P/R) was used to measure the angle of the bionic mechanical ankle. A miniature pressure sensor that is JLBS-M2 (range: 50 kg, sensitivity: 1~2 mV/V, produced by Bengbu Sensor Systems Engineering Co., Ltd, Bengbu, China) was used to measure the force and thus the output torque obtained. The resistance at room temperature of each set of SMA wires is 11.5 Ω. The current heating and data processing methods are similar to the previous experiments.

### 4.2. Angle of the Bionic Mechanical Ankle

As we mentioned above, the AMM has four SDSs which are arranged in series. To explore the dorsiflexion angle $\theta_{1}$ and plantarflexion angle $\theta_{2}$ which are shown in Figure 6a, experiments of the bionic mechanical ankle actuated by different number of SDSs were carried out. These experiments were conducted without extra loads under a pulse input with an invariable duration of the heating process that is 300 ms in each cycle. The remaining time of 6 s in each cycle is the cooling time. The heating voltage was 14 V throughout the experiments. 

**Figure 7:**
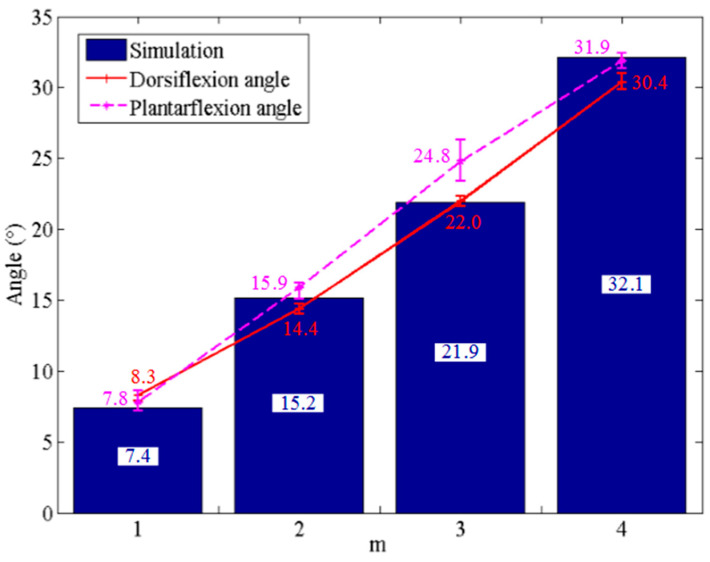
Angle of the bionic mechanical ankle actuated by different number of SDSs (*m* = 1, 2, 3 and 4).

Figure 7 shows the output angles of the bionic mechanical ankle under the driving of different number of SDSs. The simulation angles, dorsiflexion angles and plantarflexion angles under the driving of different number of SDSs are shown in Table 1. Comparing the results of the three groups, the output angles of the bionic mechanical ankle have a similar trend in the values of dorsiflexion and plantarflexion angles. We can also see that plantarflexion angle is slightly larger than dorsiflexion angle. This is perhaps because the dorsiflexion movement needs to overcome the gravity of the connecting rod that acts as the foot plate, which the plantarflexion motion does not need.

### 4.3. Torque of the Bionic Mechanical Ankle

In order to explore the torque characteristics of the bionic mechanical ankle, a series of experiments were performed. The experiments were carried out under two conditions, one for measuring the explosive output torque actuated by the short-time and large input current and the other for measuring the non-explosive output torque under normal operation. The explosive output torques were obtained under the driving of 40 V voltage with the heating time for 50 ms. Relatively, the non-explosive output torques were obtained under the driving of 14 V voltage with the heating time for 300 ms.

**Figure 8:**
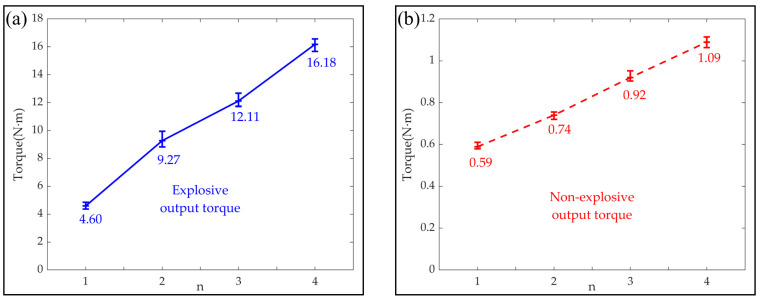
(**a**) Explosive output torque and (**b**) non-explosive output torque of the bionic mechanical ankle actuated by different number of sets of SMA wires (*n* = 1, 2, 3 and 4).

As shown in Figure 8, the explosive output torques of the bionic mechanical ankle are 4.6 Nm, 9.27 Nm, 12.11 Nm and 16.18 Nm for the number of sets of SMA wires *n* of 1, 2, 3 and 4, respectively. The non-explosive output torques of the bionic mechanical ankle are 0.59 Nm, 0.74 Nm, 0.92 Nm and 1.09 Nm for the number of sets of SMA wires *n* of 1, 2, 3 and 4, respectively.

### 4.4. Matrix Control of the Bionic Mechanical Ankle

During the temperature-induced phase transformation, the resistance of the SMA wires will change accordingly. Based on this feature, we proposed the resistance self-feedback heating strategy to adaptively regulate the heating process of the SMA wires and hence prevent their overheating in our previous work [27]. Herein, we applied the heating strategy to regulate the heating process of the AMM as well. In order to detect the resistance variation of the SMA wires, a constant sampling resistor was connected with the AMM in series. We got the instantaneous resistance by sampling the voltage across the constant resistor. 

**Figure 9:**
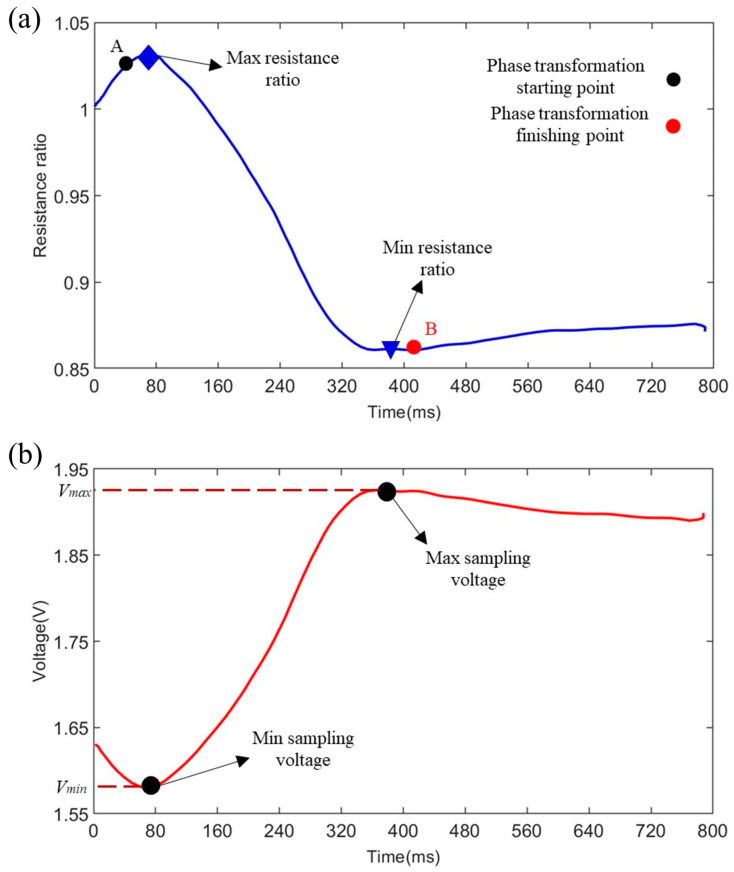
(**a**) The resistance ratio of the SMA wires and (**b**) the voltage of the sampling resistor in the heating process.

Figure 9 shows the resistance ratio of the SMA wires and the voltage of the sampling resistor during the 800 ms heating process under the heating voltage 12 V. The resistance ratio represents the ratio of the instantaneous resistance of the SMA wires to their initial resistance at room temperature. The relationship between the resistance ratio of the SMA wires and voltage of the sampling resistor follows the resistance partial pressure principle, so the minimum and maximum sampling voltages correspond to the maximum and minimum resistance ratios which are closer to the phase transformation starting point A and finishing point B, respectively. We can use the maximum and minimum ratios to determine when to switch of the input voltage. Based on this, the heating time in a selected cycle can be optimized. Hence, preventing the overheating of the SMA wires.

**Figure 10:**
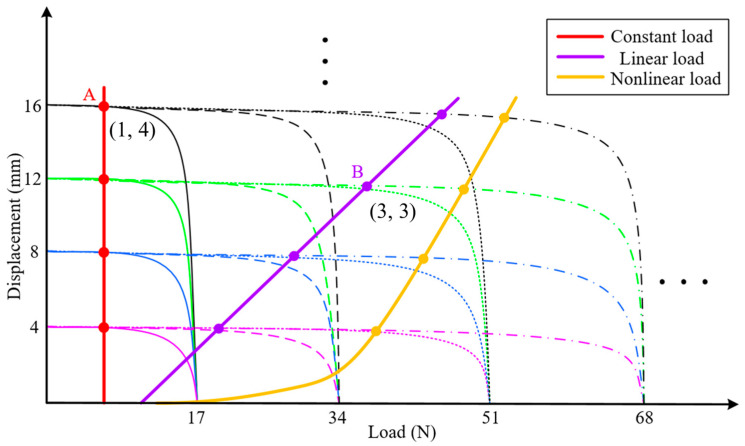
The ideal relationship between output displacement and load of the AMM. Solid, dashed, dotted and dash-dotted lines indicate that one, two, three and four SMA wires, respectively, are actuated in one SDS. Magenta, blue, green and black colors indicate that one, two, three and four SDSs, respectively, are actuated simultaneously. “…” indicate that more load-displacement combinations are possible.

**Figure 11:**
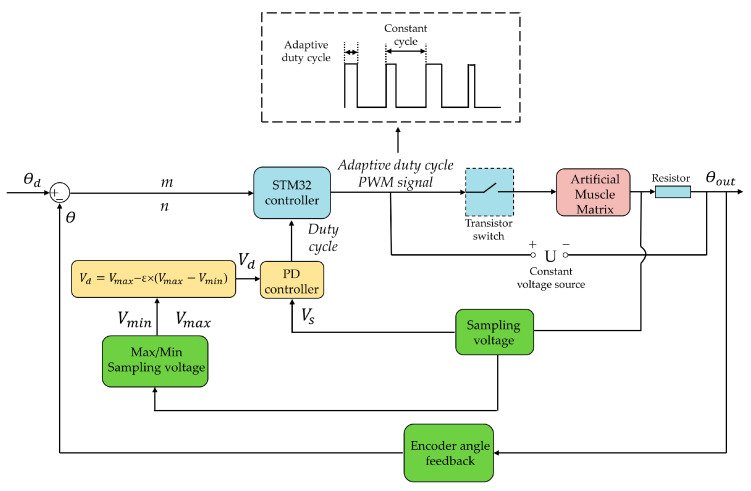
Schematic of the matrix control strategy.

In order to obtain and hold different output angles of the homemade bionic mechanical ankle under different loads, the matrix control strategy was proposed, which was based on the step output characteristic of force and displacement of AMM. The number sequence (*n*, *m*) of the series/parallel SMA wires needed to work in the AMM could be determined according to the load and displacement output requirements. *n* in the number sequence (*n*, *m*) of SMA wire determines the maximum output force that AMM can achieve, and *m* determines the maximum displacement that AMM can output. As shown in Figure 10, three different forms of external loads are defined in the figure, namely constant load, linear load and nonlinear load. Taking the working condition shown at point A of constant load as an example, the required output force can be satisfied by only one SMA wire, and the output displacement can be satisfied by four SMA wires. Therefore, the number sequence of SMA wires needed to control the work in the AMM can be obtained as (1,4). Similarly, the working sequence of SMA wire under linear load B as shown in the figure is (3,3). It can also be seen from Figure 10 that the output force is continuous but the output displacement is segmented. It is impossible to realize the displacement output of an intermediate position only by controlling the SMA wire sequence. At this point, the intermediate state can be achieved by using self-sensing method (resistance self-feedback) and PD control strategy. So, as shown in Figure 11, according to the desired angle $\theta_{d}$ of the bionic mechanical ankle, the designed number of sections *m* and the designed number of SMA wires in each section *n* can be set initially. Based on *m* and *n*, the STM32 controller send heating signals to the transistor switch whose high level (corresponding to the heating process) is variable but the cycle time is constant. The holding heating applied a PD algorithm to regulate the duty cycle of pulse-width modulation (PWM) wave which was used to heat the SMA wires of the AMM. We aim to propose the holding heating strategy is to explore a smart control method to mimic the holding motion of AMM, which can hold objects for a long time without any harm to SMA wires. The adaptive duty cycle was produced by comparing the desired sampling voltage to the sampling voltage of the constant sampling resistor.

The desired sampling voltage is given by:

(9) $$V_{d}=V_{\max}-\varepsilon \times (V_{\max}-V_{\min})$$

where $V_{d}$ represents the desired sampling voltage, ε is a coefficient at a range of [0,1] in the heating strategy, $V_{\max}$ and $V_{\min}$ represent the maximum and minimum sampling voltages as shown in Figure 9b, respectively.

**Figure 12:**
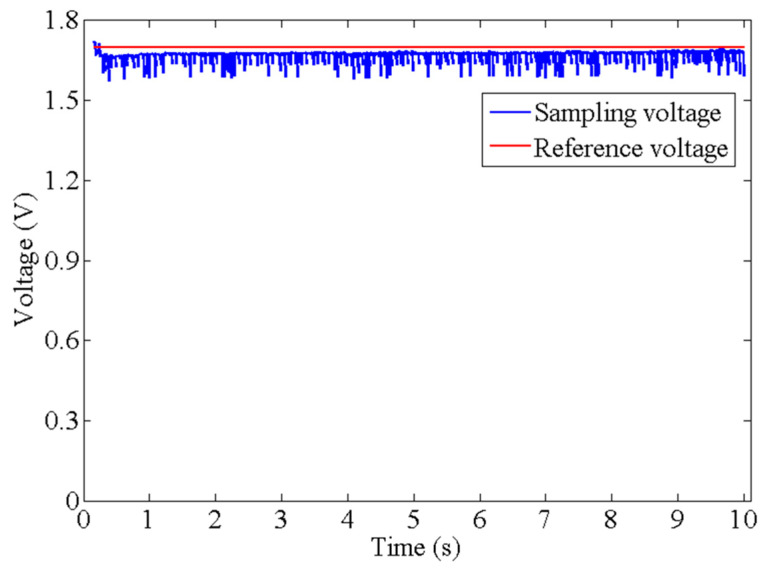
The sampling voltage and reference voltage in the holding experiment based on the PD algorithm.

Figure 12 exhibits the relationship between the reference value that is $V_{d}$ and the sampling voltage at the holding experiment in which the AMM kept the holding state at a time of 10 s. Under the PD algorithm, the sampling voltage was capable of tracing the reference value with a reasonable fluctuation. It can be seen that there is no overshoot phenomenon of the tracing voltage which has an increasing trend, but smaller than the reference value, and finally the interval value between them is steady. Although there is an interval value between the tracing and reference values, the non-overshoot enables the AMM to produce a steady holding state.

**Figure 13:**
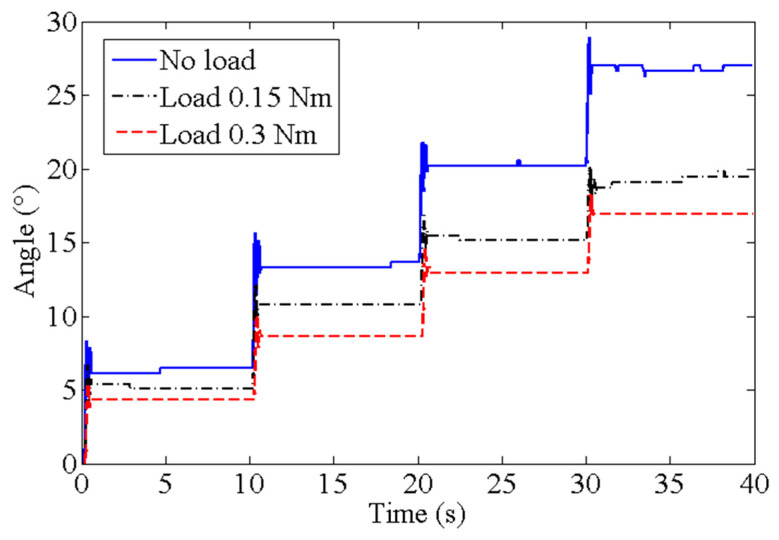
The output angles of the bionic mechanical ankle under the loads of 0 Nm, 0.15 Nm and 0.3 Nm.

**Figure 14:**
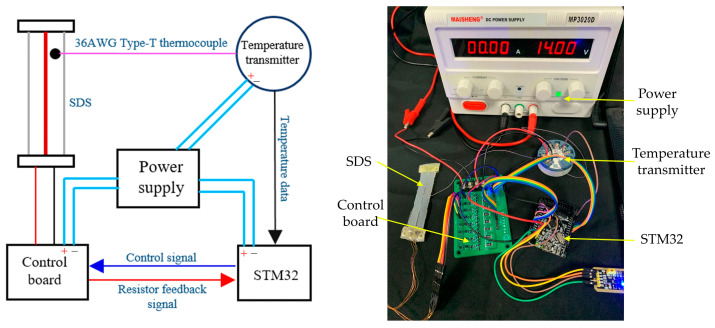
Schematic diagram and experimental device of the layout of the temperature sensor.

**Figure 15:**
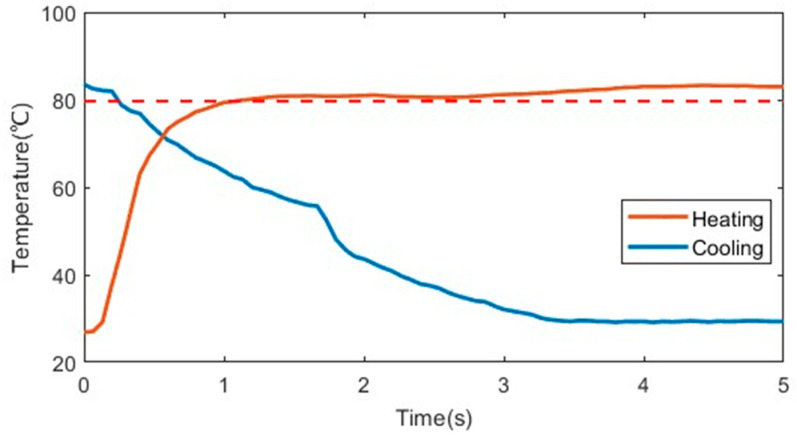
The temperature changes of the SDS in the holding experiment based on the PD algorithm.

In order to correct the output angles to the desired angles of the bionic mechanical ankle, an encoder was used to collect the rotation angles in real time. *m* and *n* was produced by comparing the desired angles to the real-time angles, as shown in Figure 11. In order to measure the output angle under different loads, we attach steel balance blocks (specification: 5–10 g) to the end of the rotating connector, and determine the applied load by calculation. The different output angles of the bionic mechanical ankle at the loads of 0 Nm, 0.15 Nm and 0.3 Nm are showed in Figure 13. When the load increases, the output angle will decrease accordingly. This is because the load will change the crystal structure of the alloy wire and affect the performance of shape memory effect, which leads to the deviation of the output angle. Every output angle kept the holding state at a time of 10 s with a reasonable fluctuation based on the PD control strategy. We can also see the obvious output angle gradient of the bionic mechanical ankle which is consistent with the experimental results in the previous chapters. Additionally, in order to verify the effectiveness of the holding heating strategy adopted in this paper, the insulated 36AWG T-type thermocouple was spot-welded to the SMA wire and cast into the SDS for temperature measurement, as shown in Figure 14. Temperature variations during both heating and cooling are illustrated in Figure 15. The orange and blue curves, respectively denote temperature changes over time during heating and cooling. The red dashed line signifies the phase transformation finish temperature of the SMA. Importantly, temperature remains relatively stable upon reaching the phase transformation finish temperature, effectively averting AMM overheating.

### 4.5. Bionic Joint Performance Comparison

Robot joint design is one of the important directions of robot research. There are many researches on bionic joint, and they all have good structure and performance. This summary summarizes the work of some existing bionic joints, whose driving bionics, maximum output angle, and non-explosive maximum output torque are shown in Table 2.

As can be seen from the table, compared with other bionic joints, the bionic joint designed in this paper adopts more advanced materials, which not only supports bidirectional rotation, but also has great advantages in terms of output torque.

## 5. Conclusions

This paper develops an SMA-based artificial muscle matrix which contains a number of SDSs in series, and each SDS is composed of many SMA wires arranged in parallel. By controlling the number of the working SDSs and the number of the working SMA wires in each SDS, we can obtain different a displacement and force output of the AMM. A simple demo version of the AMM was made which comprises 4 SDSs and 4 SMA wires in each SDS. Through the kinematics simulation and experiments, we obtained the output displacement (approx. 4 mm) of the SDS and bearing capacity (about 12 N) of one SMA wire and confirmed the effectiveness of the matrix control strategy to achieve force and displacement output independently and controllably. The demo version of the AMM was used to a bionic mechanical ankle to demonstrate the actuating capability of the AMM. Experimental results showed that the angle and force of the bionic mechanical ankle were output independently and had a significant gradient. In addition, by using the self-sensing method (resistance self-feedback) which is proposed in our previous work and PD control strategy, the output angle and force of the bionic mechanical ankle can be maintained for a long time without overheating of the AMM.

## Figures and Tables

**Table 1:** Output angle under the driving of different number of SDSs.

Driving Number of SDSs	Simulation Angles	Dorsiflexion Angles	Plantarflexion Angles
1	7.4°	8.3°	7.8°
2	15.2°	14.4°	15.9°
3	21.9°	22.0°	24.8°
4	32.1°	30.4°	31.9°

**Table 2:** Parameter performance of each bionic joint.

Model	Driving Mode	Bidirectional Rotation	Maximum Output Angles	Maximum Output Torque
Hao [31]	PAM	No	119.7°	-
Cui [32]	Motor	Yes	125°	0.58 Nm
Lohse [33]	SMA	No	56.6°	-
This paper	SMA	Yes	62.3°	1.09 Nm

## Data Availability

Data are contained within the article.

## Appendix A

**Table A1:** Parameters in the model of the SDS.

Description (Parameter)	Value (Unit)	Description (Parameter)	Value (Unit)
Number of the SMA wires ( $n_{1}$ )	8	Martensitic transformation start temperature ( ${T_{s}}^{M}$ )	54.5 °C
Diameter of the SMA wires ( $d_{sma}$ )	0.15 mm	Martensitic transformation finish temperature ( ${T_{f}}^{M}$ )	41.4 °C
Length of the SMA wires ( $L_{0}$ )	80 mm	Austenitic Young’s modulus ( $E_{A}$ )	83 GPa
Resistance per meter ( μ )	57 Ω/m	Martensitic Young’s modulus ( $E_{M}$ )	28 GPa
Density of the SMA wires ( $\rho_{sma}$ )	6.45 g/cm^3^	Maximum residual strain ( $\varepsilon_{r}$ )	5%
Specific heat capacity ( $c_{sma}$ )	837 J/(kg·°C)	Effect of stress on austenitic transformation ( $C_{A}$ )	12 MPa/°C
Latent heat of transformation ( H )	24.2 × 10^3^ J/kg	Effect of stress on martensitic transformation ( $C_{M}$ )	10 MPa/°C
Supply current ( $i_{sma}$ )	1.2 A	SMA initial martensite fraction ( $\xi_{0}$ )	1
Austenite transformation start temperature ( ${T_{s}}^{A}$ )	71.5 °C	Spring stiffness ( k )	500 N/m
Austenite transformation finish temperature ( ${T_{f}}^{A}$ )	79.7 °C	Ambient temperature ( $T_{0}$ )	25 °C
